# Supplementary material for: From sequence to enzyme mechanism using multi-label machine learning
Source: BMC Bioinformatics. 2014 May 19;15:150. doi: 10.1186/1471-2105-15-150 (PMC4229970; doi:10.1186/1471-2105-15-150)
Supplement: Additional file 2 — Java code of ml2db. Additional file ml2db_code.tar.gz contains the Java source code to run the multi-label machine learning experiments and save the results to database. The code’s Javadoc is included. [file 1471-2105-15-150-S2.zip › additional file 2/ml2db/utils/doc/test/database/DbConnPropsTest.html]

DbConnPropsTest


---


|  |  |  |
| --- | --- | --- |
| **Overview** \| **Package** \| **Class** \| **Use** \| **Tree** \| **Deprecated** \| **Index** \| **Help** | |  |
| PREV CLASS   **NEXT CLASS** | **FRAMES**    **NO FRAMES**     **All Classes** |  |
| SUMMARY: NESTED \| FIELD \| CONSTR \| METHOD | DETAIL: FIELD \| CONSTR \| METHOD |  |


---


## test.database Class DbConnPropsTest

```
java.lang.Object
  junit.framework.Assert
      junit.framework.TestCase
          test.database.DbConnPropsTest
```

**All Implemented Interfaces:**: junit.framework.Test

---

``` public class DbConnPropsTest extends junit.framework.TestCase ```

Class

**Version:**
:   27 Apr 2010

**Author:**
:   Luna De Ferrari luna.deferrari-at-ed.ac.uk

---

**Constructor Summary**

`DbConnPropsTest()`


| **Method Summary** | |
| --- | --- |
| `static DbConn` | `getDbConnProps()` |
| `void` | `setUp()`             the database connection properties file for test (database = test) |
| `void` | `tearDown()` |
| `void` | `testGetConnection()` |
| `void` | `testGetConnectionLog()` |
| `void` | `testGetDatabaseName()` |
| `void` | `testGetDatabaseType()` |
| `void` | `testGetDriverName()` |
| `void` | `testGetHost()` |
| `void` | `testGetStatement()` |

**Methods inherited from class junit.framework.TestCase**

`countTestCases, getName, run, run, runBare, setName, toString`

**Methods inherited from class junit.framework.Assert**

`assertEquals, assertEquals, assertEquals, assertEquals, assertEquals, assertEquals, assertEquals, assertEquals, assertEquals, assertEquals, assertEquals, assertEquals, assertEquals, assertEquals, assertEquals, assertEquals, assertEquals, assertEquals, assertEquals, assertEquals, assertFalse, assertFalse, assertNotNull, assertNotNull, assertNotSame, assertNotSame, assertNull, assertNull, assertSame, assertSame, assertTrue, assertTrue, fail, fail, failNotEquals, failNotSame, failSame, format`

**Methods inherited from class java.lang.Object**

`equals, getClass, hashCode, notify, notifyAll, wait, wait, wait`

**Constructor Detail**

### DbConnPropsTest

```
public DbConnPropsTest()
```


**Method Detail**

### getDbConnProps

```
public static DbConn getDbConnProps()
```

---


### setUp

```
public void setUp()
           throws java.lang.Exception
```

:   the database connection properties file for test (database = test)

    :   **Overrides:**: `setUp` in class `junit.framework.TestCase`
    :   **Throws:**: `java.lang.Exception`

---


### tearDown

```
public void tearDown()
              throws java.lang.Exception
```

:   **Overrides:**: `tearDown` in class `junit.framework.TestCase`
:   **Throws:**: `java.lang.Exception`

---


### testGetConnection

```
public void testGetConnection()
```

---


### testGetConnectionLog

```
public void testGetConnectionLog()
```

---


### testGetDatabaseName

```
public void testGetDatabaseName()
```

---


### testGetDatabaseType

```
public void testGetDatabaseType()
```

---


### testGetDriverName

```
public void testGetDriverName()
```

---


### testGetHost

```
public void testGetHost()
```

---


### testGetStatement

```
public void testGetStatement()
```


---


|  |  |  |
| --- | --- | --- |
| **Overview** \| **Package** \| **Class** \| **Use** \| **Tree** \| **Deprecated** \| **Index** \| **Help** | |  |
| PREV CLASS   **NEXT CLASS** | **FRAMES**    **NO FRAMES**     **All Classes** |  |
| SUMMARY: NESTED \| FIELD \| CONSTR \| METHOD | DETAIL: FIELD \| CONSTR \| METHOD |  |


---
